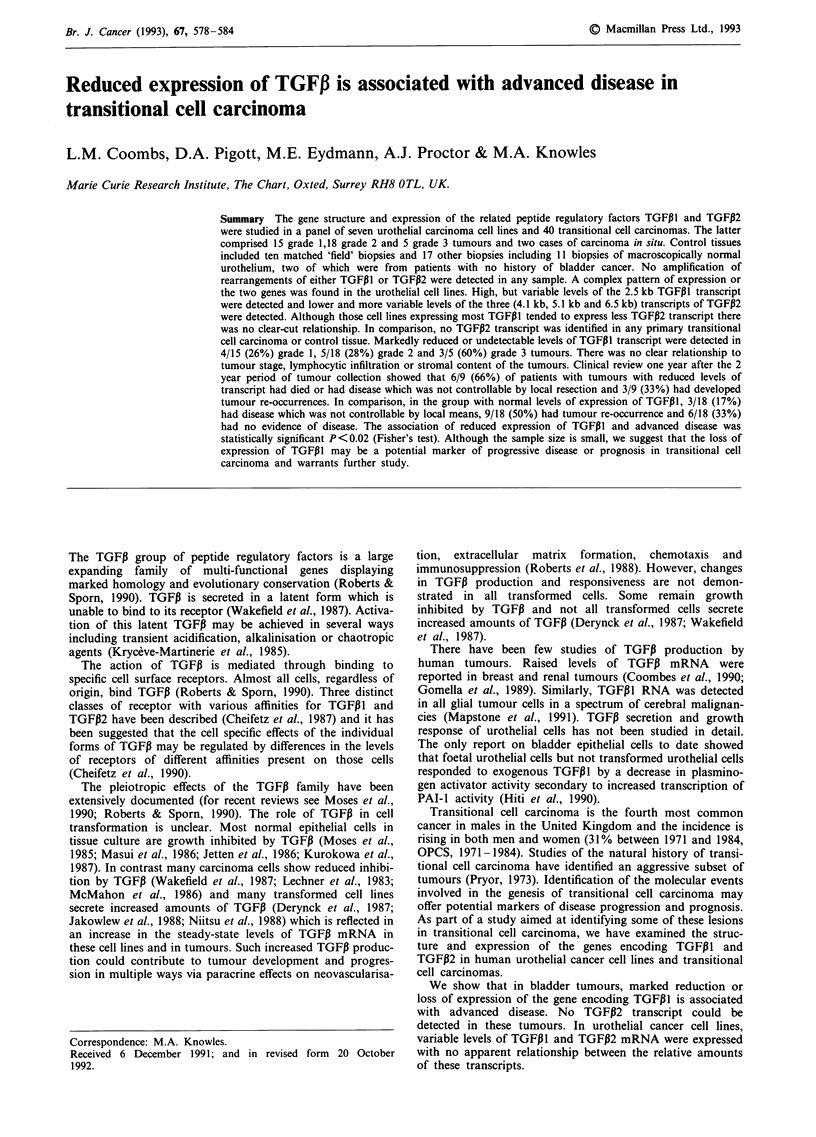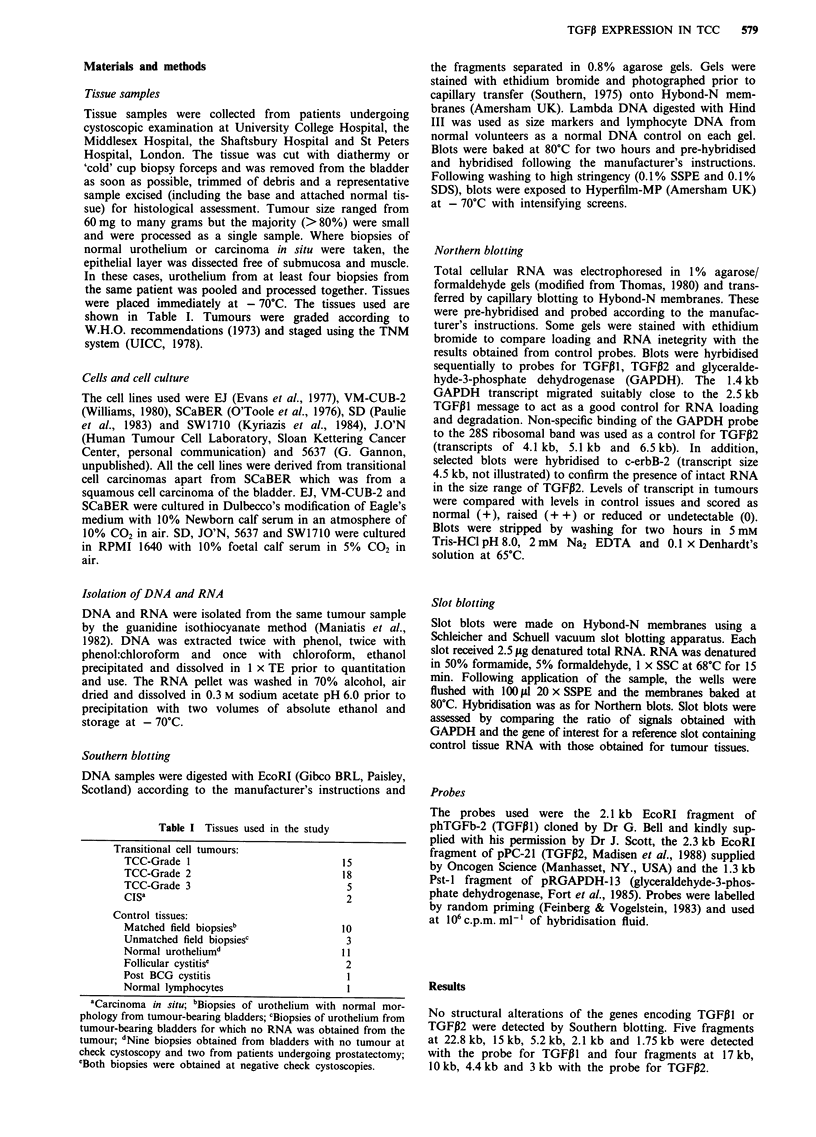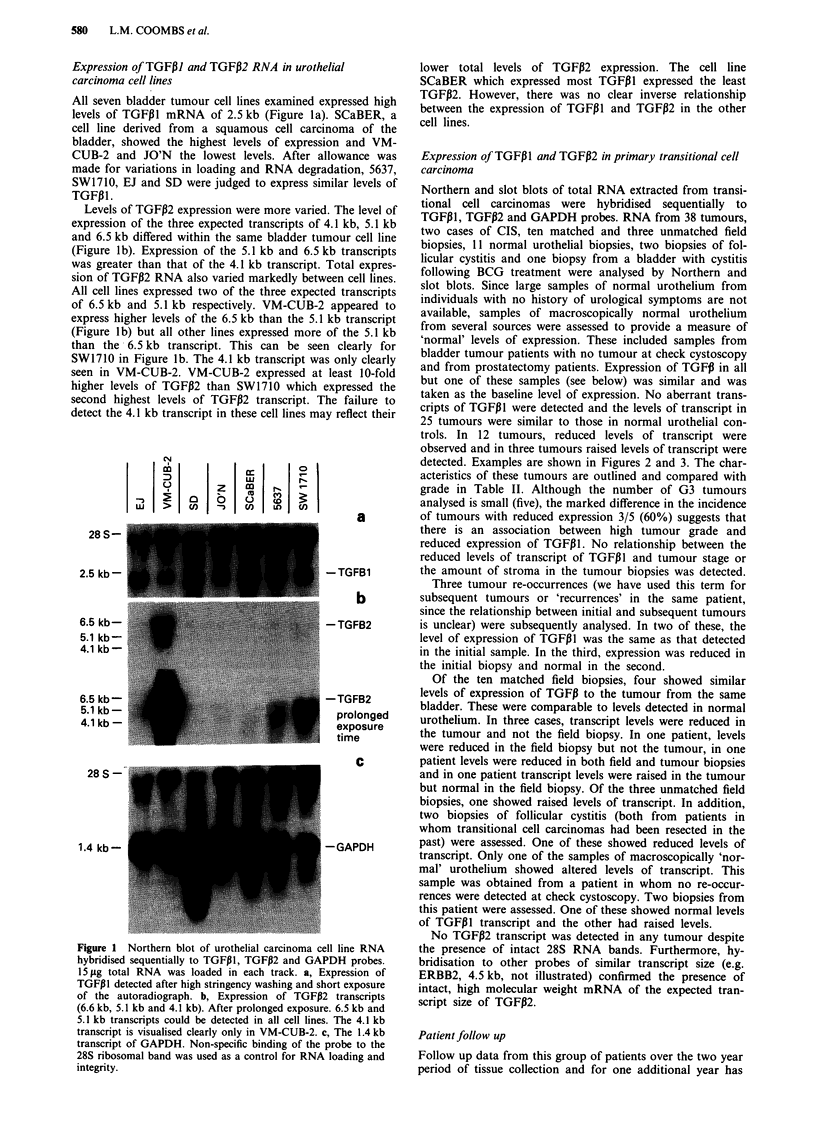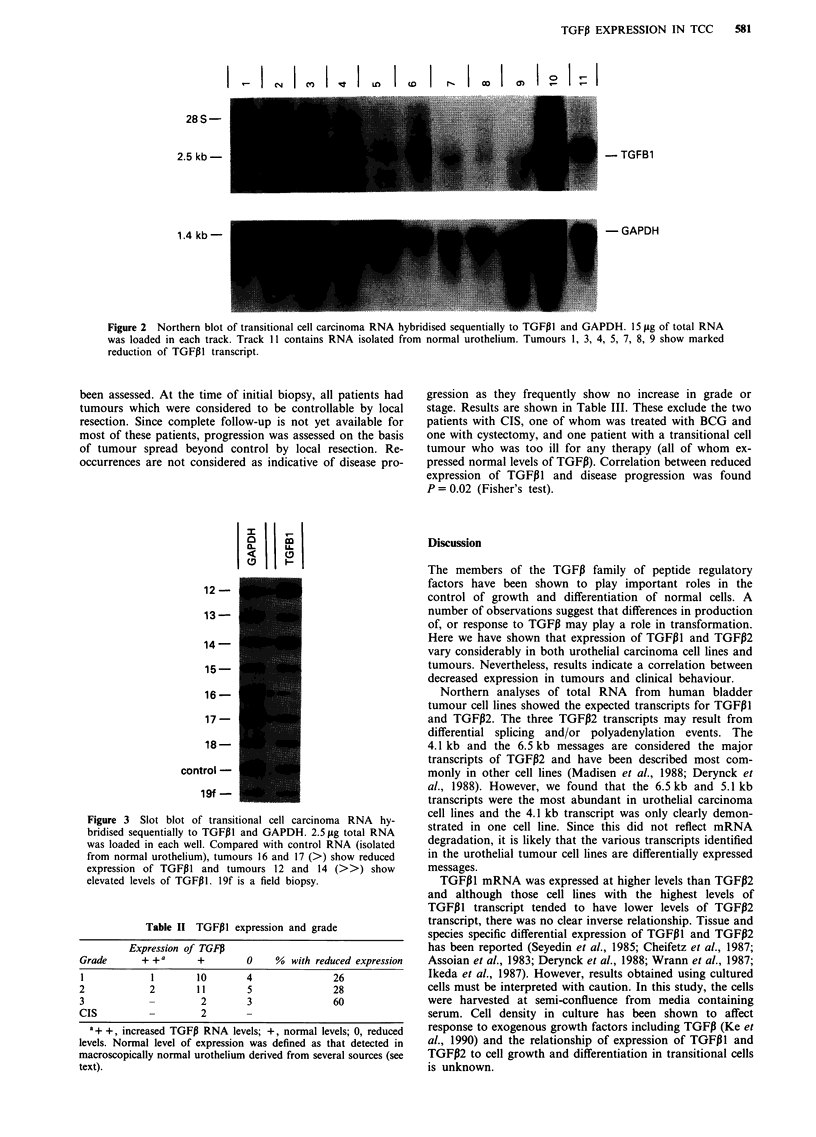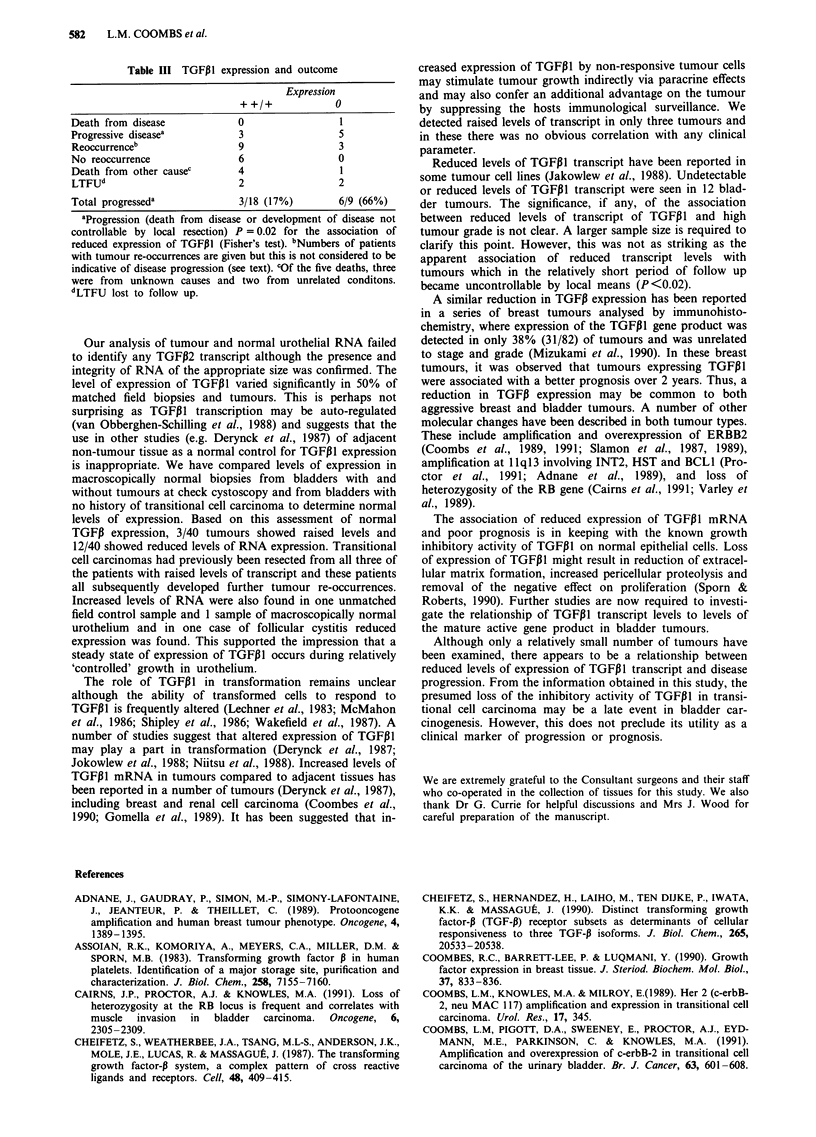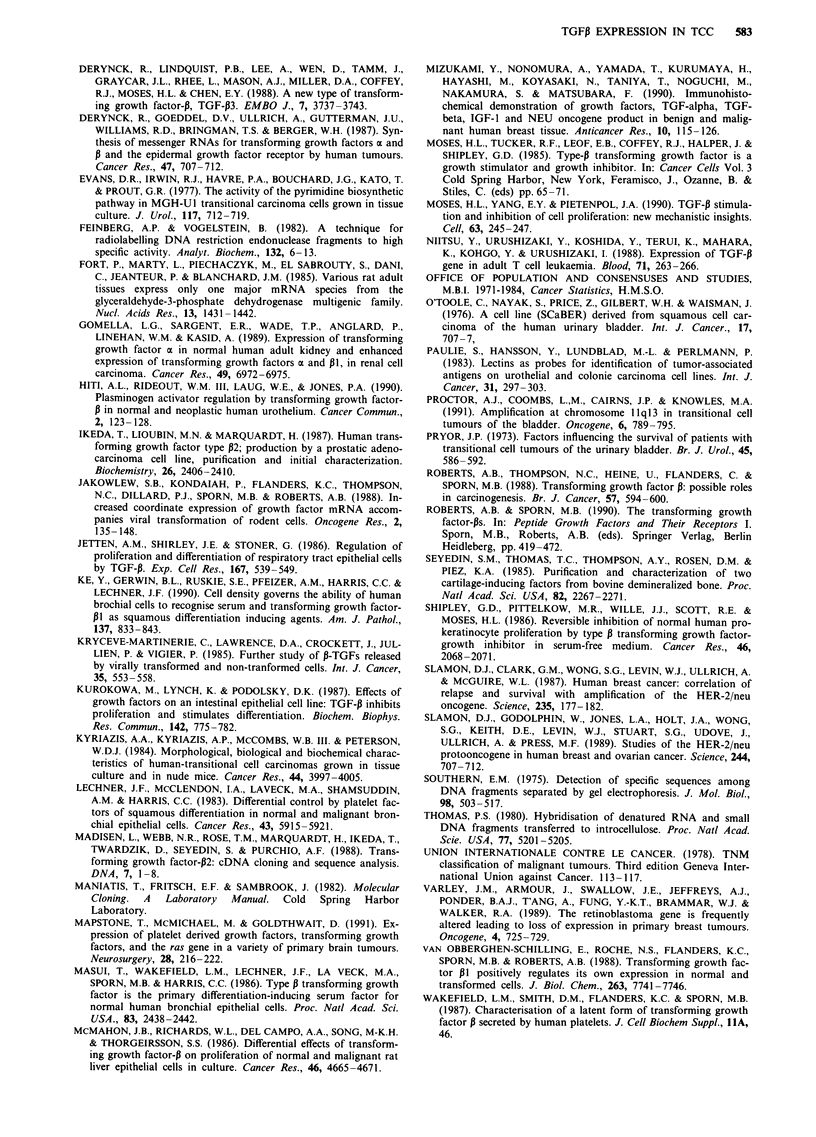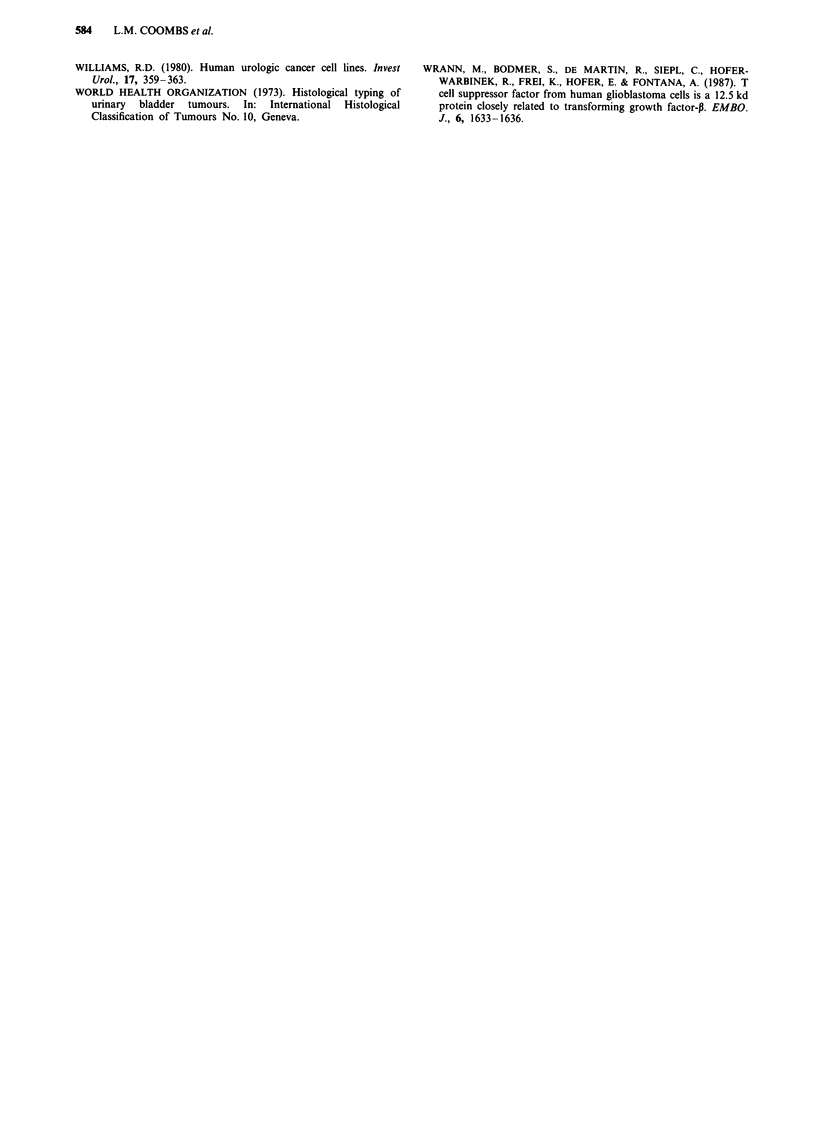# Reduced expression of TGF beta is associated with advanced disease in transitional cell carcinoma.

**DOI:** 10.1038/bjc.1993.106

**Published:** 1993-03

**Authors:** L. M. Coombs, D. A. Pigott, M. E. Eydmann, A. J. Proctor, M. A. Knowles

**Affiliations:** Marie Curie Research Institute, Oxted, Surrey, UK.

## Abstract

**Images:**


 © Macmillan Press Ltd., 1993

### Reduced expression of TGFβ is associated with advanced disease in transitional cell carcinoma

L.M. Coombs, D.A. Pigott, M.E. Eydmann, A.J. Proctor & M.A. Knowles

*Marie Curie Research Institute, The Chart, Oxted, Surrey RH8 0TL, UK.*

**Summary** The gene structure and expression of the related peptide regulatory factors TGFβ1 and TGFβ2 were studied in a panel of seven urothelial carcinoma cell lines and 40 transitional cell carcinomas. The latter comprised 15 grade 1,18 grade 2 and 5 grade 3 tumours and two cases of carcinoma *in situ*. Control tissues included ten matched 'field' biopsies and 17 other biopsies including 11 biopsies of macroscopically normal urothelium, two of which were from patients with no history of bladder cancer. No amplification of rearrangements of either TGFβ1 or TGFβ2 were detected in any sample. A complex pattern of expression or the two genes was found in the urothelial cell lines. High, but variable levels of the 2.5 kb TGFβ1 transcript were detected and lower and more variable levels of the three (4.1 kb, 5.1 kb and 6.5 kb) transcripts of TGFβ2 were detected. Although those cell lines expressing most TGFβ1 tended to express less TGFβ2 transcript there was no clear-cut relationship. In comparison, no TGFβ2 transcript was identified in any primary transitional cell carcinoma or control tissue. Markedly reduced or undetectable levels of TGFβ1 transcript were detected in 4/15 (26%) grade 1, 5/18 (28%) grade 2 and 3/5 (60%) grade 3 tumours. There was no clear relationship to tumour stage, lymphocytic infiltration or stromal content of the tumours. Clinical review one year after the 2 year period of tumour collection showed that 6/9 (66%) of patients with tumours with reduced levels of transcript had died or had disease which was not controllable by local resection and 3/9 (33%) had developed tumour re-occurrences. In comparison, in the group with normal levels of expression of TGFβ1, 3/18 (17%) had disease which was not controllable by local means, 9/18 (50%) had tumour re-occurrence and 6/18 (33%) had no evidence of disease. The association of reduced expression of TGFβ1 and advanced disease was statistically significant $P < 0.02$ (Fisher's test). Although the sample size is small, we suggest that the loss of expression of TGFβ1 may be a potential marker of progressive disease or prognosis in transitional cell carcinoma and warrants further study.

The TGFβ group of peptide regulatory factors is a large expanding family of multi-functional genes displaying marked homology and evolutionary conservation (Roberts & Sporn, 1990). TGFβ is secreted in a latent form which is unable to bind to its receptor (Wakefield *et al.*, 1987). Activation of this latent TGFβ may be achieved in several ways including transient acidification, alkalinisation or chaotropic agents (Kryçève-Martinerie *et al.*, 1985).

The action of TGFβ is mediated through binding to specific cell surface receptors. Almost all cells, regardless of origin, bind TGFβ (Roberts & Sporn, 1990). Three distinct classes of receptor with various affinities for TGFβ1 and TGFβ2 have been described (Cheifetz *et al.*, 1987) and it has been suggested that the cell specific effects of the individual forms of TGFβ may be regulated by differences in the levels of receptors of different affinities present on those cells (Cheifetz *et al.*, 1990).

The pleiotropic effects of the TGFβ family have been extensively documented (for recent reviews see Moses *et al.*, 1990; Roberts & Sporn, 1990). The role of TGFβ in cell transformation is unclear. Most normal epithelial cells in tissue culture are growth inhibited by TGFβ (Moses *et al.*, 1985; Masui *et al.*, 1986; Jetten *et al.*, 1986; Kurokowa *et al.*, 1987). In contrast many carcinoma cells show reduced inhibition by TGFβ (Wakefield *et al.*, 1987; Lechner *et al.*, 1983; McMahon *et al.*, 1986) and many transformed cell lines secrete increased amounts of TGFβ (Derynck *et al.*, 1987; Jakowlew *et al.*, 1988; Niitsu *et al.*, 1988) which is reflected in an increase in the steady-state levels of TGFβ mRNA in these cell lines and in tumours. Such increased TGFβ production could contribute to tumour development and progression in multiple ways via paracrine effects on neovascularisation, extracellular matrix formation, chemotaxis and immunosuppression (Roberts *et al.*, 1988). However, changes in TGFβ production and responsiveness are not demonstrated in all transformed cells. Some remain growth inhibited by TGFβ and not all transformed cells secrete increased amounts of TGFβ (Derynck *et al.*, 1987; Wakefield *et al.*, 1987).

There have been few studies of TGFβ production by human tumours. Raised levels of TGFβ mRNA were reported in breast and renal tumours (Coombes *et al.*, 1990; Gomella *et al.*, 1989). Similarly, TGFβ1 RNA was detected in all glial tumour cells in a spectrum of cerebral malignancies (Mapstone *et al.*, 1991). TGFβ secretion and growth response of urothelial cells has not been studied in detail. The only report on bladder epithelial cells to date showed that foetal urothelial cells but not transformed urothelial cells responded to exogenous TGFβ1 by a decrease in plasminogen activator activity secondary to increased transcription of PAI-1 activity (Hiti *et al.*, 1990).

Transitional cell carcinoma is the fourth most common cancer in males in the United Kingdom and the incidence is rising in both men and women (31% between 1971 and 1984, OPCS, 1971–1984). Studies of the natural history of transitional cell carcinoma have identified an aggressive subset of tumours (Pryor, 1973). Identification of the molecular events involved in the genesis of transitional cell carcinoma may offer potential markers of disease progression and prognosis. As part of a study aimed at identifying some of these lesions in transitional cell carcinoma, we have examined the structure and expression of the genes encoding TGFβ1 and TGFβ2 in human urothelial cancer cell lines and transitional cell carcinomas.

We show that in bladder tumours, marked reduction or loss of expression of the gene encoding TGFβ1 is associated with advanced disease. No TGFβ2 transcript could be detected in these tumours. In urothelial cancer cell lines, variable levels of TGFβ1 and TGFβ2 mRNA were expressed with no apparent relationship between the relative amounts of these transcripts.

Correspondence: M.A. Knowles.
Received 6 December 1991; and in revised form 20 October 1992.

### Materials and methods

### *Tissue samples*

Tissue samples were collected from patients undergoing cystoscopic examination at University College Hospital, the Middlesex Hospital, the Shaftsbury Hospital and St Peters Hospital, London. The tissue was cut with diathermy or 'cold' cup biopsy forceps and was removed from the bladder as soon as possible, trimmed of debris and a representative sample excised (including the base and attached normal tissue) for histological assessment. Tumour size ranged from 60 mg to many grams but the majority (> 80%) were small and were processed as a single sample. Where biopsies of normal urothelium or carcinoma *in situ* were taken, the epithelial layer was dissected free of submucosa and muscle. In these cases, urothelium from at least four biopsies from the same patient was pooled and processed together. Tissues were placed immediately at − 70°C. The tissues used are shown in Table I. Tumours were graded according to W.H.O. recommendations (1973) and staged using the TNM system (UICC, 1978).

### *Cells and cell culture*

The cell lines used were EJ (Evans *et al.*, 1977), VM-CUB-2 (Williams, 1980), SCaBER (O'Toole *et al.*, 1976), SD (Paulie *et al.*, 1983) and SW1710 (Kyriazis *et al.*, 1984), J.O'N (Human Tumour Cell Laboratory, Sloan Kettering Cancer Center, personal communication) and 5637 (G. Gannon, unpublished). All the cell lines were derived from transitional cell carcinomas apart from SCaBER which was from a squamous cell carcinoma of the bladder. EJ, VM-CUB-2 and SCaBER were cultured in Dulbecco's modification of Eagle's medium with 10% Newborn calf serum in an atmosphere of 10% $CO_2$ in air. SD, JO'N, 5637 and SW1710 were cultured in RPMI 1640 with 10% foetal calf serum in 5% $CO_2$ in air.

### *Isolation of DNA and RNA*

DNA and RNA were isolated from the same tumour sample by the guanidine isothiocyanate method (Maniatis *et al.*, 1982). DNA was extracted twice with phenol, twice with phenol:chloroform and once with chloroform, ethanol precipitated and dissolved in 1 × TE prior to quantitation and use. The RNA pellet was washed in 70% alcohol, air dried and dissolved in 0.3 M sodium acetate pH 6.0 prior to precipitation with two volumes of absolute ethanol and storage at − 70°C.

### *Southern blotting*

DNA samples were digested with EcoRI (Gibco BRL, Paisley, Scotland) according to the manufacturer's instructions and the fragments separated in 0.8% agarose gels. Gels were stained with ethidium bromide and photographed prior to capillary transfer (Southern, 1975) onto Hybond-N membranes (Amersham UK). Lambda DNA digested with Hind III was used as size markers and lymphocyte DNA from normal volunteers as a normal DNA control on each gel. Blots were baked at 80°C for two hours and pre-hybridised and hybridised following the manufacturer's instructions. Following washing to high stringency (0.1% SSPE and 0.1% SDS), blots were exposed to Hyperfilm-MP (Amersham UK) at − 70°C with intensifying screens.

**Table I** Tissues used in the study

| | |
|---|---|
| Transitional cell tumours: | |
| TCC-Grade 1 | 15 |
| TCC-Grade 2 | 18 |
| TCC-Grade 3 | 5 |
| CIS[a] | 2 |
| Control tissues: | |
| Matched field biopsies[b] | 10 |
| Unmatched field biopsies[c] | 3 |
| Normal urothelium[d] | 11 |
| Follicular cystitis[e] | 2 |
| Post BCG cystitis | 1 |
| Normal lymphocytes | 1 |

[a]Carcinoma *in situ*; [b]Biopsies of urothelium with normal morphology from tumour-bearing bladders; [c]Biopsies of urothelium from tumour-bearing bladders for which no RNA was obtained from the tumour; [d]Nine biopsies obtained from bladders with no tumour at check cystoscopy and two from patients undergoing prostatectomy; [e]Both biopsies were obtained at negative check cystoscopies.

### *Northern blotting*

Total cellular RNA was electrophoresed in 1% agarose/formaldehyde gels (modified from Thomas, 1980) and transferred by capillary blotting to Hybond-N membranes. These were pre-hybridised and probed according to the manufacturer's instructions. Some gels were stained with ethidium bromide to compare loading and RNA inetegrity with the results obtained from control probes. Blots were hyrbidised sequentially to probes for TGFβ1, TGFβ2 and glyceraldehyde-3-phosphate dehydrogenase (GAPDH). The 1.4 kb GAPDH transcript migrated suitably close to the 2.5 kb TGFβ1 message to act as a good control for RNA loading and degradation. Non-specific binding of the GAPDH probe to the 28S ribosomal band was used as a control for TGFβ2 (transcripts of 4.1 kb, 5.1 kb and 6.5 kb). In addition, selected blots were hybridised to c-erbB-2 (transcript size 4.5 kb, not illustrated) to confirm the presence of intact RNA in the size range of TGFβ2. Levels of transcript in tumours were compared with levels in control issues and scored as normal (+), raised (+ +) or reduced or undetectable (0). Blots were stripped by washing for two hours in 5 mM Tris-HCl pH 8.0, 2 mM $Na_2$ EDTA and 0.1 × Denhardt's solution at 65°C.

### *Slot blotting*

Slot blots were made on Hybond-N membranes using a Schleicher and Schuell vacuum slot blotting apparatus. Each slot received 2.5 μg denatured total RNA. RNA was denatured in 50% formamide, 5% formaldehyde, 1 × SSC at 68°C for 15 min. Following application of the sample, the wells were flushed with 100 μl 20 × SSPE and the membranes baked at 80°C. Hybridisation was as for Northern blots. Slot blots were assessed by comparing the ratio of signals obtained with GAPDH and the gene of interest for a reference slot containing control tissue RNA with those obtained for tumour tissues.

### *Probes*

The probes used were the 2.1 kb EcoRI fragment of phTGFb-2 (TGFβ1) cloned by Dr G. Bell and kindly supplied with his permission by Dr J. Scott, the 2.3 kb EcoRI fragment of pPC-21 (TGFβ2, Madisen *et al.*, 1988) supplied by Oncogen Science (Manhasset, NY., USA) and the 1.3 kb Pst-1 fragment of pRGAPDH-13 (glyceraldehyde-3-phosphate dehydrogenase, Fort *et al.*, 1985). Probes were labelled by random priming (Feinberg & Vogelstein, 1983) and used at $10^6$ c.p.m. $ml^{-1}$ of hybridisation fluid.

### Results

No structural alterations of the genes encoding TGFβ1 or TGFβ2 were detected by Southern blotting. Five fragments at 22.8 kb, 15 kb, 5.2 kb, 2.1 kb and 1.75 kb were detected with the probe for TGFβ1 and four fragments at 17 kb, 10 kb, 4.4 kb and 3 kb with the probe for TGFβ2.

*Expression of* TGFβ1 *and* TGFβ2 *RNA in urothelial carcinoma cell lines*

All seven bladder tumour cell lines examined expressed high levels of TGFβ1 mRNA of 2.5 kb (Figure 1a). SCaBER, a cell line derived from a squamous cell carcinoma of the bladder, showed the highest levels of expression and VM-CUB-2 and JO'N the lowest levels. After allowance was made for variations in loading and RNA degradation, 5637, SW1710, EJ and SD were judged to express similar levels of TGFβ1.

Levels of TGFβ2 expression were more varied. The level of expression of the three expected transcripts of 4.1 kb, 5.1 kb and 6.5 kb differed within the same bladder tumour cell line (Figure 1b). Expression of the 5.1 kb and 6.5 kb transcripts was greater than that of the 4.1 kb transcript. Total expression of TGFβ2 RNA also varied markedly between cell lines. All cell lines expressed two of the three expected transcripts of 6.5 kb and 5.1 kb respectively. VM-CUB-2 appeared to express higher levels of the 6.5 kb than the 5.1 kb transcript (Figure 1b) but all other lines expressed more of the 5.1 kb than the 6.5 kb transcript. This can be seen clearly for SW1710 in Figure 1b. The 4.1 kb transcript was only clearly seen in VM-CUB-2. VM-CUB-2 expressed at least 10-fold higher levels of TGFβ2 than SW1710 which expressed the second highest levels of TGFβ2 transcript. The failure to detect the 4.1 kb transcript in these cell lines may reflect their lower total levels of TGFβ2 expression. The cell line SCaBER which expressed most TGFβ1 expressed the least TGFβ2. However, there was no clear inverse relationship between the expression of TGFβ1 and TGFβ2 in the other cell lines.

**Figure 1** Northern blot of urothelial carcinoma cell line RNA hybridised sequentially to TGFβ1, TGFβ2 and GAPDH probes. 15 μg total RNA was loaded in each track. **a**, Expression of TGFβ1 detected after high stringency washing and short exposure of the autoradiograph. **b**, Expression of TGFβ2 transcripts (6.6 kb, 5.1 kb and 4.1 kb). After prolonged exposure. 6.5 kb and 5.1 kb transcripts could be detected in all cell lines. The 4.1 kb transcript is visualised clearly only in VM-CUB-2. **c**, The 1.4 kb transcript of GAPDH. Non-specific binding of the probe to the 28S ribosomal band was used as a control for RNA loading and integrity.

*Expression of* TGFβ1 *and* TGFβ2 *in primary transitional cell carcinoma*

Northern and slot blots of total RNA extracted from transitional cell carcinomas were hybridised sequentially to TGFβ1, TGFβ2 and GAPDH probes. RNA from 38 tumours, two cases of CIS, ten matched and three unmatched field biopsies, 11 normal urothelial biopsies, two biopsies of follicular cystitis and one biopsy from a bladder with cystitis following BCG treatment were analysed by Northern and slot blots. Since large samples of normal urothelium from individuals with no history of urological symptoms are not available, samples of macroscopically normal urothelium from several sources were assessed to provide a measure of 'normal' levels of expression. These included samples from bladder tumour patients with no tumour at check cystoscopy and from prostatectomy patients. Expression of TGFβ in all but one of these samples (see below) was similar and was taken as the baseline level of expression. No aberrant transcripts of TGFβ1 were detected and the levels of transcript in 25 tumours were similar to those in normal urothelial controls. In 12 tumours, reduced levels of transcript were observed and in three tumours raised levels of transcript were detected. Examples are shown in Figures 2 and 3. The characteristics of these tumours are outlined and compared with grade in Table II. Although the number of G3 tumours analysed is small (five), the marked difference in the incidence of tumours with reduced expression 3/5 (60%) suggests that there is an association between high tumour grade and reduced expression of TGFβ1. No relationship between the reduced levels of transcript of TGFβ1 and tumour stage or the amount of stroma in the tumour biopsies was detected.

Three tumour re-occurrences (we have used this term for subsequent tumours or 'recurrences' in the same patient, since the relationship between initial and subsequent tumours is unclear) were subsequently analysed. In two of these, the level of expression of TGFβ1 was the same as that detected in the initial sample. In the third, expression was reduced in the initial biopsy and normal in the second.

Of the ten matched field biopsies, four showed similar levels of expression of TGFβ to the tumour from the same bladder. These were comparable to levels detected in normal urothelium. In three cases, transcript levels were reduced in the tumour and not the field biopsy. In one patient, levels were reduced in the field biopsy but not the tumour, in one patient levels were reduced in both field and tumour biopsies and in one patient transcript levels were raised in the tumour but normal in the field biopsy. Of the three unmatched field biopsies, one showed raised levels of transcript. In addition, two biopsies of follicular cystitis (both from patients in whom transitional cell carcinomas had been resected in the past) were assessed. One of these showed reduced levels of transcript. Only one of the samples of macroscopically 'normal' urothelium showed altered levels of transcript. This sample was obtained from a patient in whom no re-occurrences were detected at check cystoscopy. Two biopsies from this patient were assessed. One of these showed normal levels of TGFβ1 transcript and the other had raised levels.

No TGFβ2 transcript was detected in any tumour despite the presence of intact 28S RNA bands. Furthermore, hybridisation to other probes of similar transcript size (e.g. ERBB2, 4.5 kb, not illustrated) confirmed the presence of intact, high molecular weight mRNA of the expected transcript size of TGFβ2.

*Patient follow up*

Follow up data from this group of patients over the two year period of tissue collection and for one additional year has

**Figure 2** Northern blot of transitional cell carcinoma RNA hybridised sequentially to TGFβ1 and GAPDH. 15 μg of total RNA was loaded in each track. Track 11 contains RNA isolated from normal urothelium. Tumours 1, 3, 4, 5, 7, 8, 9 show marked reduction of TGFβ1 transcript.

been assessed. At the time of initial biopsy, all patients had tumours which were considered to be controllable by local resection. Since complete follow-up is not yet available for most of these patients, progression was assessed on the basis of tumour spread beyond control by local resection. Re-occurrences are not considered as indicative of disease progression as they frequently show no increase in grade or stage. Results are shown in Table III. These exclude the two patients with CIS, one of whom was treated with BCG and one with cystectomy, and one patient with a transitional cell tumour who was too ill for any therapy (all of whom expressed normal levels of TGFβ). Correlation between reduced expression of TGFβ1 and disease progression was found $P = 0.02$ (Fisher's test).

**Figure 3** Slot blot of transitional cell carcinoma RNA hybridised sequentially to TGFβ1 and GAPDH. 2.5 μg total RNA was loaded in each well. Compared with control RNA (isolated from normal urothelium), tumours 16 and 17 (>) show reduced expression of TGFβ1 and tumours 12 and 14 (>>) show elevated levels of TGFβ1. 19f is a field biopsy.

**Table II** TGFβ1 expression and grade

| | Expression of TGFβ | | | |
|---|---|---|---|---|
| *Grade* | + +[a] | + | 0 | % with reduced expression |
| 1 | 1 | 10 | 4 | 26 |
| 2 | 2 | 11 | 5 | 28 |
| 3 | – | 2 | 3 | 60 |
| CIS | – | 2 | – | |

[a]+ +, increased TGFβ RNA levels; +, normal levels; 0, reduced levels. Normal level of expression was defined as that detected in macroscopically normal urothelium derived from several sources (see text).

### Discussion

The members of the TGFβ family of peptide regulatory factors have been shown to play important roles in the control of growth and differentiation of normal cells. A number of observations suggest that differences in production of, or response to TGFβ may play a role in transformation. Here we have shown that expression of TGFβ1 and TGFβ2 vary considerably in both urothelial carcinoma cell lines and tumours. Nevertheless, results indicate a correlation between decreased expression in tumours and clinical behaviour.

Northern analyses of total RNA from human bladder tumour cell lines showed the expected transcripts for TGFβ1 and TGFβ2. The three TGFβ2 transcripts may result from differential splicing and/or polyadenylation events. The 4.1 kb and the 6.5 kb messages are considered the major transcripts of TGFβ2 and have been described most commonly in other cell lines (Madisen *et al.*, 1988; Derynck *et al.*, 1988). However, we found that the 6.5 kb and 5.1 kb transcripts were the most abundant in urothelial carcinoma cell lines and the 4.1 kb transcript was only clearly demonstrated in one cell line. Since this did not reflect mRNA degradation, it is likely that the various transcripts identified in the urothelial tumour cell lines are differentially expressed messages.

TGFβ1 mRNA was expressed at higher levels than TGFβ2 and although those cell lines with the highest levels of TGFβ1 transcript tended to have lower levels of TGFβ2 transcript, there was no clear inverse relationship. Tissue and species specific differential expression of TGFβ1 and TGFβ2 has been reported (Seyedin *et al.*, 1985; Cheifetz *et al.*, 1987; Assoian *et al.*, 1983; Derynck *et al.*, 1988; Wrann *et al.*, 1987; Ikeda *et al.*, 1987). However, results obtained using cultured cells must be interpreted with caution. In this study, the cells were harvested at semi-confluence from media containing serum. Cell density in culture has been shown to affect response to exogenous growth factors including TGFβ (Ke *et al.*, 1990) and the relationship of expression of TGFβ1 and TGFβ2 to cell growth and differentiation in transitional cells is unknown.

Table III TGFβ1 expression and outcome

| | Expression | |
|---|---|---|
| | ++/+ | 0 |
| Death from disease | 0 | 1 |
| Progressive disease[a] | 3 | 5 |
| Reoccurrence[b] | 9 | 3 |
| No reoccurrence | 6 | 0 |
| Death from other cause[c] | 4 | 1 |
| LTFU[d] | 2 | 2 |
| Total progressed[a] | 3/18 (17%) | 6/9 (66%) |

[a]Progression (death from disease or development of disease not controllable by local resection) $P = 0.02$ for the association of reduced expression of TGFβ1 (Fisher's test). [b]Numbers of patients with tumour re-occurrences are given but this is not considered to be indicative of disease progression (see text). [c]Of the five deaths, three were from unknown causes and two from unrelated conditons. [d]LTFU lost to follow up.

Our analysis of tumour and normal urothelial RNA failed to identify any TGFβ2 transcript although the presence and integrity of RNA of the appropriate size was confirmed. The level of expression of TGFβ1 varied significantly in 50% of matched field biopsies and tumours. This is perhaps not surprising as TGFβ1 transcription may be auto-regulated (van Obberghen-Schilling *et al.*, 1988) and suggests that the use in other studies (e.g. Derynck *et al.*, 1987) of adjacent non-tumour tissue as a normal control for TGFβ1 expression is inappropriate. We have compared levels of expression in macroscopically normal biopsies from bladders with and without tumours at check cystoscopy and from bladders with no history of transitional cell carcinoma to determine normal levels of expression. Based on this assessment of normal TGFβ expression, 3/40 tumours showed raised levels and 12/40 showed reduced levels of RNA expression. Transitional cell carcinomas had previously been resected from all three of the patients with raised levels of transcript and these patients all subsequently developed further tumour re-occurrences. Increased levels of RNA were also found in one unmatched field control sample and 1 sample of macroscopically normal urothelium and in one case of follicular cystitis reduced expression was found. This supported the impression that a steady state of expression of TGFβ1 occurs during relatively 'controlled' growth in urothelium.

The role of TGFβ1 in transformation remains unclear although the ability of transformed cells to respond to TGFβ1 is frequently altered (Lechner *et al.*, 1983; McMahon *et al.*, 1986; Shipley *et al.*, 1986; Wakefield *et al.*, 1987). A number of studies suggest that altered expression of TGFβ1 may play a part in transformation (Derynck *et al.*, 1987; Jokowlew *et al.*, 1988; Niitsu *et al.*, 1988). Increased levels of TGFβ1 mRNA in tumours compared to adjacent tissues has been reported in a number of tumours (Derynck *et al.*, 1987), including breast and renal cell carcinoma (Coombes *et al.*, 1990; Gomella *et al.*, 1989). It has been suggested that increased expression of TGFβ1 by non-responsive tumour cells may stimulate tumour growth indirectly via paracrine effects and may also confer an additional advantage on the tumour by suppressing the hosts immunological surveillance. We detected raised levels of transcript in only three tumours and in these there was no obvious correlation with any clinical parameter.

Reduced levels of TGFβ1 transcript have been reported in some tumour cell lines (Jakowlew *et al.*, 1988). Undetectable or reduced levels of TGFβ1 transcript were seen in 12 bladder tumours. The significance, if any, of the association between reduced levels of transcript of TGFβ1 and high tumour grade is not clear. A larger sample size is required to clarify this point. However, this was not as striking as the apparent association of reduced transcript levels with tumours which in the relatively short period of follow up became uncontrollable by local means ($P<0.02$).

A similar reduction in TGFβ expression has been reported in a series of breast tumours analysed by immunohistochemistry, where expression of the TGFβ1 gene product was detected in only 38% (31/82) of tumours and was unrelated to stage and grade (Mizukami *et al.*, 1990). In these breast tumours, it was observed that tumours expressing TGFβ1 were associated with a better prognosis over 2 years. Thus, a reduction in TGFβ expression may be common to both aggressive breast and bladder tumours. A number of other molecular changes have been described in both tumour types. These include amplification and overexpression of ERBB2 (Coombs *et al.*, 1989, 1991; Slamon *et al.*, 1987, 1989), amplification at 11q13 involving INT2, HST and BCL1 (Proctor *et al.*, 1991; Adnane *et al.*, 1989), and loss of heterozygosity of the RB gene (Cairns *et al.*, 1991; Varley *et al.*, 1989).

The association of reduced expression of TGFβ1 mRNA and poor prognosis is in keeping with the known growth inhibitory activity of TGFβ1 on normal epithelial cells. Loss of expression of TGFβ1 might result in reduction of extracellular matrix formation, increased pericellular proteolysis and removal of the negative effect on proliferation (Sporn & Roberts, 1990). Further studies are now required to investigate the relationship of TGFβ1 transcript levels to levels of the mature active gene product in bladder tumours.

Although only a relatively small number of tumours have been examined, there appears to be a relationship between reduced levels of expression of TGFβ1 transcript and disease progression. From the information obtained in this study, the presumed loss of the inhibitory activity of TGFβ1 in transitional cell carcinoma may be a late event in bladder carcinogenesis. However, this does not preclude its utility as a clinical marker of progression or prognosis.

We are extremely grateful to the Consultant surgeons and their staff who co-operated in the collection of tissues for this study. We also thank Dr G. Currie for helpful discussions and Mrs J. Wood for careful preparation of the manuscript.

### References

ADNANE, J., GAUDRAY, P., SIMON, M.-P., SIMONY-LAFONTAINE, J., JEANTEUR, P. & THEILLET, C. (1989). Protooncogene amplification and human breast tumour phenotype. *Oncogene*, **4**, 1389–1395.

ASSOIAN, R.K., KOMORIYA, A., MEYERS, C.A., MILLER, D.M. & SPORN, M.B. (1983). Transforming growth factor β in human platelets. Identification of a major storage site, purification and characterization. *J. Biol. Chem.*, **258**, 7155–7160.

CAIRNS, J.P., PROCTOR, A.J. & KNOWLES, M.A. (1991). Loss of heterozygosity at the RB locus is frequent and correlates with muscle invasion in bladder carcinoma. *Oncogene*, **6**, 2305–2309.

CHEIFETZ, S., WEATHERBEE, J.A., TSANG, M.L-S., ANDERSON, J.K., MOLE, J.E., LUCAS, R. & MASSAGUÉ, J. (1987). The transforming growth factor-β system, a complex pattern of cross reactive ligands and receptors. *Cell*, **48**, 409–415.

CHEIFETZ, S., HERNANDEZ, H., LAIHO, M., TEN DIJKE, P., IWATA, K.K. & MASSAGUÉ, J. (1990). Distinct transforming growth factor-β (TGF-β) receptor subsets as determinants of cellular responsiveness to three TGF-β isoforms. *J. Biol. Chem.*, **265**, 20533–20538.

COOMBES, R.C., BARRETT-LEE, P. & LUQMANI, Y. (1990). Growth factor expression in breast tissue. *J. Steriod. Biochem. Mol. Biol.*, **37**, 833–836.

COOMBS, L.M., KNOWLES, M.A. & MILROY, E.(1989). Her 2 (c-erbB-2, neu MAC 117) amplification and expression in transitional cell carcinoma. *Urol. Res.*, **17**, 345.

COOMBS, L.M, PIGOTT, D.A., SWEENEY, E., PROCTOR, A.J., EYDMANN, M.E., PARKINSON, C. & KNOWLES, M.A. (1991). Amplification and overexpression of c-erbB-2 in transitional cell carcinoma of the urinary bladder. *Br. J. Cancer*, **63**, 601–608.

DERYNCK, R., LINDQUIST, P.B., LEE, A., WEN, D., TAMM, J., GRAYCAR, J.L., RHEE, L., MASON, A.J., MILLER, D.A., COFFEY, R.J., MOSES, H.L. & CHEN, E.Y. (1988). A new type of transforming growth factor-β, TGF-β3. *EMBO J.*, **7**, 3737–3743.

DERYNCK, R., GOEDDEL, D.V., ULLRICH, A., GUTTERMAN, J.U., WILLIAMS, R.D., BRINGMAN, T.S. & BERGER, W.H. (1987). Synthesis of messenger RNAs for transforming growth factors α and β and the epidermal growth factor receptor by human tumours. *Cancer Res.*, **47**, 707–712.

EVANS, D.R., IRWIN, R.J., HAVRE, P.A., BOUCHARD, J.G., KATO, T. & PROUT, G.R. (1977). The activity of the pyrimidine biosynthetic pathway in MGH-U1 transitional carcinoma cells grown in tissue culture. *J. Urol.*, **117**, 712–719.

FEINBERG, A.P. & VOGELSTEIN, B. (1982). A technique for radiolabelling DNA restriction endonuclease fragments to high specific activity. *Analyt. Biochem.*, **132**, 6–13.

FORT, P., MARTY, L., PIECHACZYK, M., EL SABROUTY, S., DANI, C., JEANTEUR, P. & BLANCHARD, J.M. (1985). Various rat adult tissues express only one major mRNA species from the glyceraldehyde-3-phosphate dehydrogenase multigenic family. *Nucl. Acids Res.*, **13**, 1431–1442.

GOMELLA, L.G., SARGENT, E.R., WADE, T.P., ANGLARD, P., LINEHAN, W.M. & KASID, A. (1989). Expression of transforming growth factor α in normal human adult kidney and enhanced expression of transforming growth factors α and β1, in renal cell carcinoma. *Cancer Res.*, **49**, 6972–6975.

HITI, A.L., RIDEOUT, W.M. III, LAUG, W.E., & JONES, P.A. (1990). Plasminogen activator regulation by transforming growth factor-β in normal and neoplastic human urothelium. *Cancer Commun.*, **2**, 123–128.

IKEDA, T., LIOUBIN, M.N. & MARQUARDT, H. (1987). Human transforming growth factor type β2; production by a prostatic adenocarcinoma cell line, purification and initial characterization. *Biochemistry*, **26**, 2406–2410.

JAKOWLEW, S.B., KONDAIAH, P., FLANDERS, K.C., THOMPSON, N.C., DILLARD, P.J., SPORN, M.B. & ROBERTS, A.B. (1988). Increased coordinate expression of growth factor mRNA accompanies viral transformation of rodent cells. *Oncogene Res.*, **2**, 135–148.

JETTEN, A.M., SHIRLEY, J.E. & STONER, G. (1986). Regulation of proliferation and differentiation of respiratory tract epithelial cells by TGF-β. *Exp. Cell Res.*, **167**, 539–549.

KE, Y., GERWIN, B.L., RUSKIE, S.E., PFEIZER, A.M., HARRIS, C.C. & LECHNER, J.F. (1990). Cell density governs the ability of human brochial cells to recognise serum and transforming growth factor-β1 as squamous differentiation inducing agents. *Am. J. Pathol.*, **137**, 833–843.

KRYCEVE-MARTINERIE, C., LAWRENCE, D.A., CROCKETT, J., JULLIEN, P. & VIGIER, P. (1985). Further study of β-TGFs released by virally transformed and non-tranformed cells. *Int. J. Cancer*, **35**, 553–558.

KUROKOWA, M., LYNCH, K. & PODOLSKY, D.K. (1987). Effects of growth factors on an intestinal epithelial cell line: TGF-β inhibits proliferation and stimulates differentiation. *Biochem. Biophys. Res. Commun.*, **142**, 775–782.

KYRIAZIS, A.A., KYRIAZIS, A.P., MCCOMBS, W.B. III. & PETERSON, W.D.J. (1984). Morphological, biological and biochemical characteristics of human-transitional cell carcinomas grown in tissue culture and in nude mice. *Cancer Res.*, **44**, 3997–4005.

LECHNER, J.F., MCCLENDON, I.A., LAVECK, M.A., SHAMSUDDIN, A.M. & HARRIS, C.C. (1983). Differential control by platelet factors of squamous differentiation in normal and malignant bronchial epithelial cells. *Cancer Res.*, **43**, 5915–5921.

MADISEN, L., WEBB, N.R., ROSE, T.M., MARQUARDT, H., IKEDA, T., TWARDZIK, D., SEYEDIN, S. & PURCHIO, A.F. (1988). Transforming growth factor-β2: cDNA cloning and sequence analysis. *DNA*, **7**, 1–8.

MANIATIS, T., FRITSCH, E.F. & SAMBROOK, J. (1982). *Molecular Cloning. A Laboratory Manual.* Cold Spring Harbor Laboratory.

MAPSTONE, T., MCMICHAEL, M. & GOLDTHWAIT, D. (1991). Expression of platelet derived growth factors, transforming growth factors, and the *ras* gene in a variety of primary brain tumours. *Neurosurgery*, **28**, 216–222.

MASUI, T., WAKEFIELD, L.M., LECHNER, J.F., LA VECK, M.A., SPORN, M.B. & HARRIS, C.C. (1986). Type β transforming growth factor is the primary differentiation-inducing serum factor for normal human bronchial epithelial cells. *Proc. Natl Acad. Sci. USA.*, **83**, 2438–2442.

MCMAHON, J.B., RICHARDS, W.L., DEL CAMPO, A.A., SONG, M-K.H. & THORGEIRSSON, S.S. (1986). Differential effects of transforming growth factor-β on proliferation of normal and malignant rat liver epithelial cells in culture. *Cancer Res.*, **46**, 4665–4671.

MIZUKAMI, Y., NONOMURA, A., YAMADA, T., KURUMAYA, H., HAYASHI, M., KOYASAKI, N., TANIYA, T., NOGUCHI, M., NAKAMURA, S. & MATSUBARA, F. (1990). Immunohistochemical demonstration of growth factors, TGF-alpha, TGF-beta, IGF-1 and NEU oncogene product in benign and malignant human breast tissue. *Anticancer Res.*, **10**, 115–126.

MOSES, H.L., TUCKER, R.F., LEOF, E.B., COFFEY, R.J., HALPER, J. & SHIPLEY, G.D. (1985). Type-β transforming growth factor is a growth stimulator and growth inhibitor. In: *Cancer Cells* Vol. 3 Cold Spring Harbor, New York, Feramisco, J., Ozanne, B. & Stiles, C. (eds) pp. 65–71.

MOSES, H.L., YANG, E.Y. & PIETENPOL, J.A. (1990). TGF-β stimulation and inhibition of cell proliferation: new mechanistic insights. *Cell*, **63**, 245–247.

NIITSU, Y., URUSHIZAKI, Y., KOSHIDA, Y., TERUI, K., MAHARA, K., KOHGO, Y. & URUSHIZAKI, I. (1988). Expression of TGF-β gene in adult T cell leukaemia. *Blood*, **71**, 263–266.

OFFICE OF POPULATION AND CONSENSUSES AND STUDIES, M.B.I. 1971-1984, *Cancer Statistics*, H.M.S.O.

O'TOOLE, C., NAYAK, S., PRICE, Z., GILBERT, W.H. & WAISMAN, J. (1976). A cell line (SCaBER) derived from squamous cell carcinoma of the human urinary bladder. *Int. J. Cancer.*, **17**, 707–7,

PAULIE, S., HANSSON, Y., LUNDBLAD, M.-L. & PERLMANN, P. (1983). Lectins as probes for identification of tumor-associated antigens on urothelial and colonie carcinoma cell lines. *Int. J. Cancer*, **31**, 297–303.

PROCTOR, A.J., COOMBS, L.,M., CAIRNS, J.P. & KNOWLES, M.A. (1991). Amplification at chromosome 11q13 in transitional cell tumours of the bladder. *Oncogene*, **6**, 789–795.

PRYOR, J.P. (1973). Factors influencing the survival of patients with transitional cell tumours of the urinary bladder. *Br. J. Urol.*, **45**, 586–592.

ROBERTS, A.B., THOMPSON, N.C., HEINE, U., FLANDERS, C. & SPORN, M.B. (1988). Transforming growth factor β: possible roles in carcinogenesis. *Br. J. Cancer*, **57**, 594–600.

ROBERTS, A.B. & SPORN, M.B. (1990). The transforming growth factor-βs. In: *Peptide Growth Factors and Their Receptors* I. Sporn, M.B., Roberts, A.B. (eds). Springer Verlag, Berlin Heidleberg, pp. 419–472.

SEYEDIN, S.M., THOMAS, T.C., THOMPSON, A.Y., ROSEN, D.M. & PIEZ, K.A. (1985). Purification and characterization of two cartilage-inducing factors from bovine demineralized bone. *Proc. Natl Acad. Sci. USA*, **82**, 2267–2271.

SHIPLEY, G.D., PITTELKOW, M.R., WILLE, J.J., SCOTT, R.E. & MOSES, H.L. (1986). Reversible inhibition of normal human prokeratinocyte proliferation by type β transforming growth factor-growth inhibitor in serum-free medium. *Cancer Res.*, **46**, 2068–2071.

SLAMON, D.J., CLARK, G.M., WONG, S.G., LEVIN, W.J., ULLRICH, A. & MCGUIRE, W.L. (1987). Human breast cancer: correlation of relapse and survival with amplification of the HER-2/neu oncogene. *Science*, **235**, 177–182.

SLAMON, D.J., GODOLPHIN, W., JONES, L.A., HOLT, J.A., WONG, S.G., KEITH, D.E., LEVIN, W.J., STUART, S.G., UDOVE, J., ULLRICH, A. & PRESS, M.F. (1989). Studies of the HER-2/neu protooncogene in human breast and ovarian cancer. *Science*, **244**, 707–712.

SOUTHERN, E.M. (1975). Detection of specific sequences among DNA fragments separated by gel electrophoresis. *J. Mol. Biol.*, **98**, 503–517.

THOMAS, P.S. (1980). Hybridisation of denatured RNA and small DNA fragments transferred to introcellulose. *Proc. Natl Acad. Scie. USA*, **77**, 5201–5205.

UNION INTERNATIONALE CONTRE LE CANCER. (1978). TNM classification of malignant tumours. Third edition Geneva International Union against Cancer. 113–117.

VARLEY, J.M., ARMOUR, J., SWALLOW, J.E., JEFFREYS, A.J., PONDER, B.A.J., T'ANG, A., FUNG, Y.-K.T., BRAMMAR, W.J. & WALKER, R.A. (1989). The retinoblastoma gene is frequently altered leading to loss of expression in primary breast tumours. *Oncogene*, **4**, 725–729.

VAN OBBERGHEN-SCHILLING, E., ROCHE, N.S., FLANDERS, K.C., SPORN, M.B. & ROBERTS, A.B. (1988). Transforming growth factor β1 positively regulates its own expression in normal and transformed cells. *J. Biol. Chem.*, **263**, 7741–7746.

WAKEFIELD, L.M., SMITH, D.M., FLANDERS, K.C. & SPORN, M.B. (1987). Characterisation of a latent form of transforming growth factor β secreted by human platelets. *J. Cell Biochem Suppl.*, **11A**, 46.

WILLIAMS, R.D. (1980). Human urologic cancer cell lines. *Invest Urol.*, **17**, 359–363.

WORLD HEALTH ORGANIZATION (1973). Histological typing of urinary bladder tumours. In: International Histological Classification of Tumours No. 10, Geneva.

WRANN, M., BODMER, S., DE MARTIN, R., SIEPL, C., HOFER-WARBINEK, R., FREI, K., HOFER, E. & FONTANA, A. (1987). T cell suppressor factor from human glioblastoma cells is a 12.5 kd protein closely related to transforming growth factor-β. *EMBO. J.*, **6**, 1633–1636.